# Supplementary material for: Causal Relationship between Aging and Anorexia Nervosa: A White-Matter-Microstructure-Mediated Mendelian Randomization Analysis
Source: Biomedicines. 2024 Aug 16;12(8):1874. doi: 10.3390/biomedicines12081874 (PMC11351342; doi:10.3390/biomedicines12081874)
Supplement: Supplementary file 1 [file biomedicines-12-01874-s001.zip › Supplementary Figure/Figure S8/Leaveoneout plot of MR analysis for TL to ubm-b-1681.pdf]

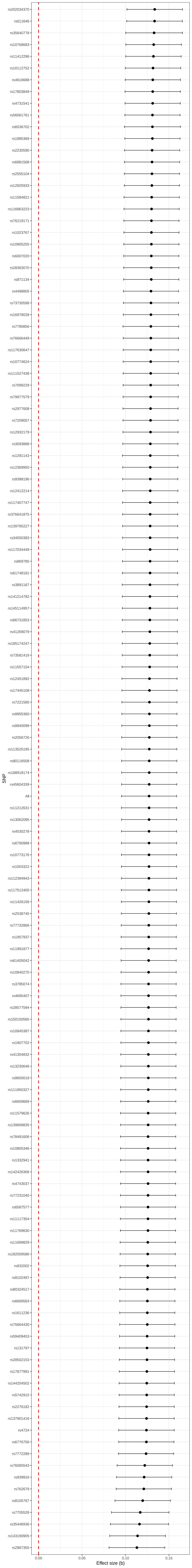
rs202034370
rs611646
rs35640778
rs10768683
rs11412296
rs10112752
rs4616688
rs17803849
rs4731541
rs56061761
rs6536702
rs1985369
rs2230590
rs6881568
rs2555104
rs12925933
rs11584821
rs116863223
rs76219171
rs1023767
rs10905255
rs6007020
rs28363070
rs871134
rs4498805
rs73730598
rs16978028
rs7790856
rs76666449
rs117630647
rs10774624
rs111527438
rs7099229
rs79977579
rs2977608
rs7209057
rs12932179
rs3093888
rs1291143
rs12369950
rs9398196
rs12412214
rs117407747
rs376641875
rs139795227
rs34550383
rs117034449
rs869785
rs61748181
rs3891167
rs141214782
rs145114957
rs66731853
rs41269079
rs185174247
rs73581419
rs11557154
rs12451892
rs17445108
rs7221585
rs9955360
rs9940099
rs2056726
rs113525195
rs80116508
rs188918174
rs45604339
All
rs11212631
rs13062095
rs4530278
rs6790988
rs10773176
rs1003322
rs112394943
rs117512405
rs11426156
rs2538745
rs77732866
rs1957937
rs11991877
rs61405042
rs10840270
rs3785074
rs4695407
rs28577594
rs150150565
rs10845387
rs1907702
rs41304832
rs13230646
rs9600019
rs111950327
rs6659669
rs11579626
rs139669835
rs78491606
rs10805346
rs1332941
rs142426306
rs4743037
rs77231040
rs6587577
rs11117354
rs11769630
rs11699829
rs182059586
rs932002
rs8102497
rs80324517
rs6669563
rs1611236
rs75664430
rs59409453
rs131797
rs28502153
rs17677991
rs144204502
rs5742915
rs2276182
rs137901416
rs4724
rs6776756
rs7772289
rs76065543
rs939916
rs762679
rs8105767
rs7705526
rs35446936
rs143190905
rs2967355
SNP
0.00
0.05
0.10
0.15
Effect size (b)
